# Gut non-bacterial microbiota contributing to alcohol-associated liver disease

**DOI:** 10.1080/19490976.2021.1984122

**Published:** 2021-10-28

**Authors:** Wenkang Gao, Yixin Zhu, Jin Ye, Huikuan Chu

**Affiliations:** a Division of Gastroenterology, Union Hospital, Tongji Medical College, Huazhong University of Science and Technology, Wuhan, China; b Department of Medicine, University of California San Diego, La Jolla, California, USA

**Keywords:** Alcohol-associated liver disease, gut-liver axis, gut barrier, liver sinusoid endothelial cells, mycobiome, CLEC7A, virome, archaeome, immune response

## Abstract

Intestinal microbiota, dominated by bacteria, plays an important role in the occurrence and the development of alcohol-associated liver disease (ALD), which is one of the most common liver diseases around the world. With sufficient studies focusing on the gut bacterial community, chronic alcohol consumption is now known as a key factor that alters the composition of gut bacterial community, increases intestinal permeability, causes intestinal dysfunction, induces bacterial translocation, and exacerbates the process of ALD via gut-liver axis. However, gut non-bacterial communities including fungi, viruses, and archaea, which may also participate in the disease, has received little attention relative to the gut bacterial community. This paper will systematically collect the latest literatures reporting non-bacterial communities in mammalian health and disease, and review their mechanisms in promoting the development of ALD including CLEC7A pathway, Candidalysin (a peptide toxin secreted by *Candida albicans*), metabolites, and other chemical substances secreted or regulated by gut commensal mycobiome, virome, and archaeome, hoping to bring novel insights on our current knowledge of ALD.

## Graphical abstract

**Figure:**
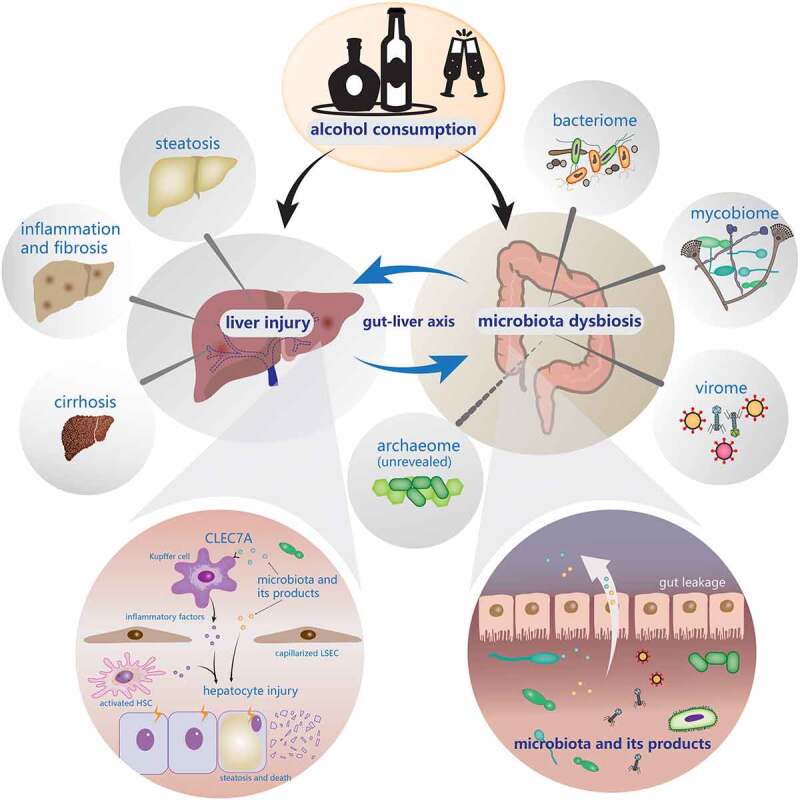

## Introduction

Alcohol-associated liver disease (ALD) is a common disease caused by alcohol use disorder (AUD), ranging from asymptomatic liver steatosis to alcohol-associated hepatitis (AH), cirrhosis, and potentially hepatocellular carcinoma (HCC). ALD is the leading indication for liver transplantation in the United States.^1,2^ Globally, about 2 million people die of liver diseases each year. In Western Europe, one-third of liver cirrhosis can be attributed to alcohol.^3^ Moreover, it has been estimated that 60–80% of liver-related deaths can be attributed to alcohol consumption.^4^ Currently, the pathogenetic mechanisms have not been fully elucidated, but they might be related to oxidative stress, acetaldehyde-induced toxicity, cytokine, and chemokine-induced inflammation.^5^ There is no effective therapeutic method for ALD till now except for liver transplantation. A great number of studies have reported that gut microbiota has an intimate relationship with ALD, which provides broader insights and opportunities for understanding and treating this disease.^6–14^ However, most studies put emphasis upon the interactions between gut bacteria and ALD, while the roles of non-bacterial communities remain unclear. Hence, this review mainly focuses on addressing the relationships between ALD and the non-bacterial communities (fungi, viruses, and archaea) in the gut, and the possible pathogenetic mechanisms mediated by those microorganisms.

## The gut-liver axis

**Figure 1.**
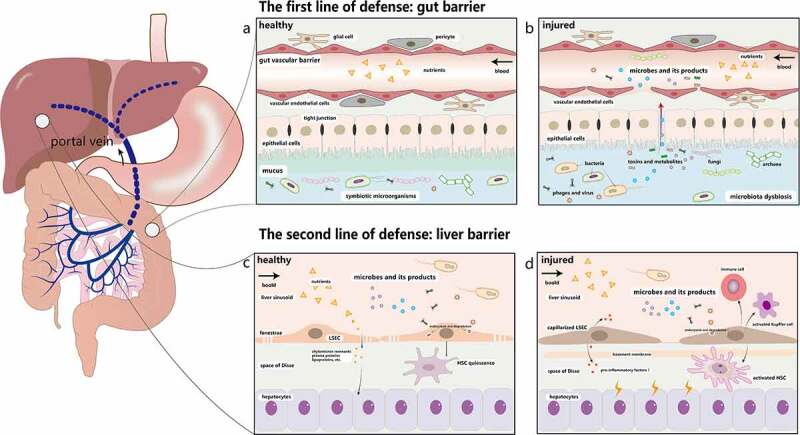
The double line of defense in the gut-liver axis. **a.** The first line of defense is the gut barrier, which is composed of the mucus barrier, the intestinal epithelial barrier, and the GVB. The mucus layer consists of a microbiota-colonized outer layer and an almost sterile inner layer. The epithelial barrier below the mucus layer is formed by epithelial cells. The deepest protective layer is the GVB. Under physiological circumstances, the healthy intestine barrier allows the absorption of nutrients while prevents the penetration of most toxic and harmful substances. **b**. Once it is damaged, intestinal microorganisms and their metabolites will move into the liver through the portal vein. **c**. LSECs are highly fenestrated cells, which can remove recycled waste products by endocytosis and degradation to protect liver against microorganisms and toxicants. They can also induce hepatic immune tolerance and keep the quiescence of HSCs and Kupffer cells. **d**. Injured LSECs become capillarized and the main characteristics of capillarization are formation of basement membrane, reduction of fenestrae and increased expression of pro-inflammatory mediators. They recruit immune cells and activate HSCs and Kupffer cells to cause liver inflammation. GVB: gut vascular barrier; LSECs: liver sinusoid endothelial cells. HSCs: hepatic stellate cells

The term gut-liver axis was first described by *Volta et al* in 1987, which pointed out the interaction between intestine and liver.^15^ During the embryonic stage, both the liver and intestine originate from the same foregut, and the two organs are anatomically connected by the portal vein system after reaching their maturity.^16^ About 70 ~ 80% of the blood from the portal vein flows to the liver,^17^ transmitting a variety of signals generated by dietary, genetic, and environmental factors.(Figure 1)^18^ As a virtual metabolic organ, the gut-liver axis achieves close-knit functional collaboration and forms a sophisticated network structure through substance metabolism, immune regulation, and interaction with the neuroendocrine system.

Normally, the healthy intestinal barrier is composed of the mucus barrier, the intestinal epithelial barrier, and the gut vascular barrier (GVB),^18–21^ which together protects the body against external insults (Figure 1(a)). In the colon, the mucus layer usually consists of a microbiota-colonized outer layer and an almost sterile inner layer. These inner and outer layers work together to provide the needed nutrients for microorganisms survival and unique niches for microorganisms inhabitation.^22,23^ Below the mucus barrier is the epithelial barrier, which is a single layer formed by the epithelial cells including enterocytes, goblet cells, tuft cells, and enterochromaffin cells.^24^ The neighboring epithelial cells also remain in close contact through tight junctions.^25–27^ Epithelial barrier’s main functions are regulating the interactions between gut microbiota and the host immune system, and selectively absorbing water, electrolytes, and nutrients.^28,29^ The GVB identified in 2015 is comprised of vascular endothelial cells, pericytes, and glial cells, which prevents the invasion of microbiota.^30,31^ As the deepest protective layer and the last defense line of the gut barrier, once the GVB is injured, the intestinal pathogens will go into the blood stream and reach other organs (Figure 1(b)). Hence, in order to fight against exogenous substances in the gut lumen, these three layers summarized above constitute the body’s first line of defense.

Once the intestinal barrier collapses, the liver becomes the first organ to encounter intestinal products, which makes it susceptible to pathological changes.^32,33^ Liver sinusoidal endothelial cells (LSECs), a major member of the hepatic barrier, provide a second line of defense against gut-derived antigens and inflammatory factors.^34^ LSECs are highly fenestrated cells (Figure 1(c)). The diameters of transcellular pores on LSECs are 50 ~ 200 nm and these LSECs usually act as a dynamic filter under physiological conditions.^35,36^ For example, small molecules, such as chylomicron remnants, plasma proteins, and lipoproteins, can cross fenestrae and reach the space of Disse for uptake and utilization by hepatocytes and HSCs.^37^ Furthermore, LSECs can remove recycled waste products and toxicants, including enteric viruses,^38,39^ bacteriophages,^40^ lipopolysaccharides (LPS),^41^ and immune complexes.^42^ LSECs can also maintain the quiescent state of hepatic stellate cells, induce hepatic immune tolerance and exert an anti-inflammatory effect.^43,44^ Due to the existence of the second line of defense, the sole injury of the intestinal barrier is unlikely to lead to significant liver damage, which has been confirmed experimentally in the previous studies.^32,33^

However, when LSECs are impaired by intestinal pathogenic factors, all the above functions are repressed. LSECs become capillarized^45^ and their phenotype is transformed into a pro-inflammatory pattern, with the formation of the basement membrane, a significant reduction of fenestrae, and increased expression of pro-inflammatory mediators such as tumor necrosis factor α (TNF-α), macrophage inflammatory protein 1 alpha (MIP-1α), monocyte chemoattractant protein-1 (MCP-1), and chemokine (C-C motif) ligand 5 (CCL5).^46^ At the same time, LSECs recruit immune cells and activate HSCs and Kupffer cells to induce liver inflammation.^47^ (Figure 1(d)). Inflammatory factors and metabolites secreted by the injured liver will further aggravate dysfunction of the intestinal barrier, creating a vicious circle eventually. In the future, understanding the mechanism of barrier damage and repair, finding damage markers that are easily detectable, developing drugs to repair the barrier are important steps for the treatment of liver and intestine-related diseases, and achieving the transformation from basic research to clinical implementations.

A complete gut-liver axis relies on not only the intact intestinal barrier and normal liver function but also the healthy gut microbiota. Gut microbiota is composed of 500 to 1000 different species belonging to more than 70 genera.^48,49^ The number of bacteria residing in the human body is close to the number of human cells.^50,51^ Not merely can they participate in digestion, absorption and metabolism of food but can also influence the intestinal structure and immune function both directly and indirectly. Therefore, clarifying the changes and roles of gut microbiota in ALD is of great importance for us to understand its pathogenesis and to develop effective treatment strategies. Whereas an immense number of reviews have covered gut bacteria in detail,^52–56^ thus we center our discussion on the gut mycobiome, virome, and archaeome in human and animal models.

## Gut mycobiome

Fungi are ubiquitous in the natural environment and are important parts of the earth’s ecosystem.^57^ The fungal community is considered as a fundamental component of the human microbiota, coexisting and interacting with other microorganisms in the gut.^58^ However, they are usually neglected in studies owing to their relatively low abundance in the human body. For instance, there have been almost 100 times more research reports on microbiota than on mycobiota from 2008 to 2018.^59^ With the increase of related research data and the development of methodology, scientists begin to realize its indispensable role in metabolism, immune regulation, and pathophysiology of the host.

### Composition and colonization

Fungi occupy a relatively small proportion of the human microbiota. Fungi comprise approximately 0.03% of the fecal microbiome.^60,61^ However, this does not mean that it is meaningless to study fungi. Conversely, some scientists contended that previous efforts might underestimate the proportion of fungi in gut microbiota owing to limited annotated reference sequences.^62^ Besides, fungal cells are generally larger than bacterial cells, indicating that fungi contain much more biomass and metabolites, which cannot be reflected by simple genome-counting.^62^ Thus, more data and further studies are required for accurately estimating the amount of living gut fungi and completing the annotated fungal genome.

The gut fungi in adults are mainly composed of three phyla, *Ascomycetes, Basidiomycetes*, and *Zygomycetes*,^63^ which are regulated by many factors such as environment,^64^ diet,^65–67^ host immunity,^68^ etc. Both human and animal studies have confirmed that the composition of the intestinal mycobiome is dynamic over time and much more mutable than the component of gut bacteria.^69,70^ A previous study indicated that intestinal fungi have colonized in human 10 days after birth.^71^ But recently, fungi were found in the first-pass meconium, suggesting that colonization of gut fungi may exist earlier than expected.^72^ In the gut of 10-day to 3-month-old infants, the fungal species that had the highest abundance are *Debaryomyces hansenii* and *Rhodotorula mucilaginosa*, while *S. cerevisiae* becomes the most abundant fungal species in the gut of babies 1–2 years after birth.^71^ From that timepoint and on, the gut fungi develops and gets closer and closer to the intestinal fungal components of the adult. Therefore, given the variability of fungal components, some scholars advocate that fungi do not colonize the gastrointestinal tract in healthy adults, and they may originate from the fungi already present in the mouth or diet, and persistent fungal colonization may be a symptom of disease.^67^ In their study, when a healthy adult volunteer increased the frequency of cleaning teeth, the abundance of *C. albicans* in stool was lowered 10-fold to 100-fold, suggesting that the oral cavity may be the primary source of *C. albicans* detected in the stool of healthy people.^67^ Indeed, existing research rarely consider the influence of oral and dietary fungi, especially in the studies of exploring changes in the gut fungi compositions. It is difficult to determine whether differences in stool fungi are due to increased transient fungi or due to real intestinal colonization. Overall, the colonization and composition of intestinal fungi are still in their infancy. In the future, further studies with a larger sample size should be done in this field, and sequencing methods with higher sensitivity and accuracy need to be invented to detect intestinal fungi at various time points of embryonic development.

### Intestinal fungi and alcohol-associated liver disease

The studies on the correlation between gut mycobiota and ALD are limited and mostly focus on the changes in fungal composition and exploration of potential pathogenic mechanisms. Previous studies reported a decrease in the diversity of mycobiota in the feces of alcohol consumers, with a significant overgrowth of *Candida*, and a decrease in *Epicoccum*, unclassified fungi, *Galactomyces*, and *Debaryomyces*.^73,74^ Similarly, recent research reported mycobiota dysbiosis with an overgrowth of *Candida* as well.^75–77^ Although there were differences in the severity of alcohol-related liver disease, there were no significant differences in the intestinal mycobiota among patients with non-progressive alcohol-associated liver disease, alcohol-associated hepatitis, and alcoholic cirrhosis.^73^

**Figure 2.**
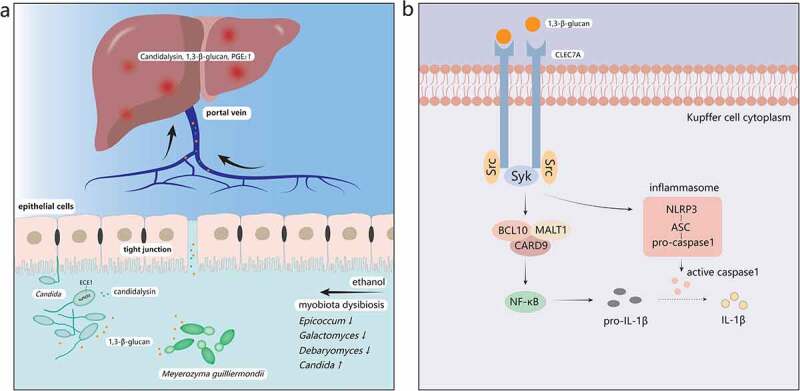
The mechanisms of ALD induced by the gut mycobiota. **a.** Ethanol abuse leads to disordered gut mycobiota. *Epicoccum, Galactomyces*, and *Debaryomyces* decrease, while *Candida* increases significantly in the intestine. 1,3-β-glucan, a main component of fungal cell walls, and Candidalysin, a fungal metabolite, translocate into the systemic circulation through the injured intestinal barrier and reach the liver first. **b**. 1,3-β-glucan binds to dectin-1 of Kupffer cells. Src kinases phosphorylate tyrosine residue of dectin-1 and recruit Syk, which can activate the CARD9/BCL10/MALT1 complex. Then NF-κB will be activated by the complex and produce pro-IL-1β. Syk also promotes the secretion of caspase-1 through the activated NLRP3/ASC/pro-caspase-1 complex. Caspase-1 then cleaves pro-IL-1β into mature IL-1β, mediating liver inflammation and damage. Syk, spleen tyrosine kinase; CARD9, caspase recruitment domain 9; BCL10, B-cell lymphoma 10; MALT1, mucosa-associated lymphoid tissue lymphoma-translocation gene 1; NF-κB, nuclear factor kappa B; NLRP3, NOD-like receptor family pyrin domain containing 3; ASC, apoptosis-associated speck-like protein containing a CARD

As for the role of gut fungi in the pathogenesis of ALD, there remain two main pathways at present: one is dependent on the C-type lectin domain family 7 member A (CLEC7A) pathway of Kupffer cells in liver and the other is independent of that pathway (Figure 2(a)).

CLEC7A (also called dectin-1) is a pattern recognition receptor primarily located on the surface of Kupffer cells and macrophages in the liver.^73^ CLEC7A is capable of recognizing 1,3-β-glucan,^78^ which is widely found in fungal cell walls, and plays an important role in resisting fungi and fungal products.(Figure 2(b))^79^ Upon ligand binding, tyrosine residue of dectin-1 is phosphorylated by Src kinases, which creates a docking site for spleen tyrosine kinase (Syk), then Syk activates the caspase recruitment domain 9 (CARD9)/B-cell lymphoma 10 (BCL10)/mucosa-associated lymphoid tissue lymphoma-translocation gene 1 (MALT1) complex, which leads to the activation of nuclear factor kappa B (NF-κB) and promotes cytokines expression including pro-interleukin-1β (pro-IL-1β).^80,81^ Furthermore, Syk can activate NOD-like receptor family pyrin domain containing 3 (NLRP3) inflammasome, which usually forms a complex with apoptosis-associated speck-like protein containing a CARD (ASC) and pro-caspase-1. ASC can activate pro-caspase-1 into caspase-1, which will cleave pro-IL-1β to produce mature IL-1β and lead to liver damage by activating downstream cascade.^82,83^ An animal study reported that the development of ALD required IL-1β which was activated via a caspase-1-dependent process, and that IL-1β signaling was essential for alcohol-induced inflammation, steatosis, liver damage, and fibrosis.^84^ A subsequent study^73^ also revealed that chronic alcohol intake could increase the number of fungi in mice intestine, and that overgrowth of fungi could produce more fungal products such as 1,3-β-glucan, which can be translocated into the liver through already dysfunctional enteric tight junctions, bind to the Dectin-1 of Kupffer cells, and finally lead to the secretion of IL-1β to promote liver inflammation. However, one study indicated that water-insoluble polysaccharides (WIP), a 1,3-β-glucan from *Wolfporia cocos*, alleviated the hepatic inflammatory injury and steatosis effectively in mice with alcoholic hepatic steatosis (AHS), which suggested the protective effect of 1,3-β-glucan in liver diseases but the mechanism needs to be further studied.^85^

Another fungus-related mechanism is mainly associated with its metabolites. As an opportunistic pathogen in the intestine, *Candida albicans* has attracted extensive attention because of its overgrowth in ALD patients.^73^ Recent studies have proven that *Candida albicans* could secrete a type of peptide toxin called Candidalysin, which directly damages epithelial membranes and activates host immune responses.^86^ One previous study found an interesting phenomenon that the number of Candidalysin positive *C. albicans* increased in patients with AH and the expression of the extent of cell elongation 1 (ECE1) gene encoding Candidalysin also increased significantly.^87^ In addition, clinical studies suggested that Candidalysin was positively associated with the severity and mortality of AH.^87^ With regard to the underlying pathogenic mechanisms of Candidalysin, however, it does not affect the intestinal permeability nor interacts with the glucan receptor CLEC7A, but it increases the expression of IL-1β, CXCL1, and CXCL2 in mice liver, which contributes to recruit immune cells and induce hepatocyte death. In brief, the specific molecular mechanisms need further research.

A recent research also found the contribution of fungi-induced metabolite – prostaglandin E_2_ (PGE_2_) to the development of ALD.^85^ They observed an overwhelming increase of the commensal fungus *Meyerozyma guilliermondii* and hepatic PGE_2_ in mice with alcoholic hepatic steatosis (AHS). By introducing antifungal agents, the level of liver PGE_2_ was significantly reduced. Interestingly, it is still unclear whether PGE_2_ is produced directly by the gut fungi or indirectly by the fungi-stimulated liver. To sum up, the concrete mechanisms of gut fungi-induced PGE_2_ in the development of ALD awaits further study.

In addition, the host immune response plays a key role in the process of ALD as well. A recent study^74^ revealed a novel finding that the level of serum anti-Saccharomyces cerevisiae antibodies (ASCA) was significantly higher in patients with AH compared with alcohol use disorder and nonalcoholic controls. As a marker of the host immune response to fungus and fungal products, ASCA was positively associated with mortality in AH patients.

In summary, it is not difficult to find that a few potential noninvasive indicators, such as the level of serum ASCA, serum Candidalysin, serum 1,3-β-glucan, and the expression of ECE1 in stool, can help us determine the progress or prognosis of ALD. In addition, inhibiting the increase of alcohol-related gut fungi, especially some specific antifungal drugs against harmful mycobiome, may be a promising therapeutic method for ALD, but its clinical application has yet to be confirmed in large-scale cohort studies. Also, further compelling investigations should explore the causal relationship between the specific fungus strain isolated from the gut and the pathogenesis of ALD.

### Probiotic fungi

In 2014, the International Scientific Association of Probiotics and Prebiotics (ISAPP) defined probiotics as “live microorganisms that, when administered in adequate amounts, confer a health benefit on the host”.^88^ There are many types of probiotics, such as *Bifidobacterium bifidus, Lactobacillus*, and *Saccharomyces boulardii*, of which *S. boulardii* is probably the most commonly used probiotic fungi. Animal studies have confirmed that *S. boulardii* could change gut microbiota and attenuate liver injury, inflammation,^89–91^ and fibrosis,^92^ potentially indicating its protective and therapeutic role in liver diseases. A recent study revealed that most strains of *Saccharomyces* and *non-Saccharomyces* yeasts evaluated in their work are safe microorganisms, and those could be regarded as a valid alternative to the widely available probiotic yeast *S. boulardii*.^93^ However, some side effects of *S. boulardii*, mainly presented as fungemia, were reported in clinical application, which are usually found in the elderly, immunosuppressed, and patients with broad-spectrum antibiotics.^94–97^ Furthermore, macrofungi with large sporocarps or fruiting bodies, also known as mushrooms, particularly showed their medicinal properties.^98^ Some types of macrofungi including *Agaricus bisporus*^99–101^ and *Pleurotus ostreatus*^102^ also possess hepato-protective activity. In conclusion, supplementing probiotic fungi to prevent or treat liver diseases can be expected in the future.

## Gut virome

### Composition and distribution

Viruses have their own important roles in the intestine. As the most numerous biological entities in nature,^103,104^ viruses are composed of endogenous retroviruses, eukaryotic viruses, and bacteriophages. Most viruses in the human body reside in the gastrointestinal tract.

The gut viruses, even called virus fingerprints, are unique to each person and mainly consist of bacteriophages.^105^ However, identifying viral sequences in large and mixed gut microbiota is extremely challenging because viruses lack a universal viral marker.^106^ Thus, an overwhelming number of gut bacteriophages remain uncultured and unclassified till now, and their specific hosts and infection strategies have not been clarified either. Scientists also named gut viruses “the dark matter” in the intestine, which showed that relevant research on them were far from enough.^107–109^

Fortunately, the Gut Virome Database (GVD)^110^ and the Gut Phage Database^111^ have been established and are continuously updating, which will advance research of gut virome considerably. According to the GVD,^110^ 97.7% of viral populations are phages, 2.1% are eukaryotic viruses, and 0.1% are archaeal viruses. About 10^15^ bacteriophages settle in a healthy human intestine, which is 10 times the number of symbiotic bacteria.^112^ The main phages come from the order *Caudovirales* including *Siphoviridae, Myoviridae*, and *Podoviridae*.^113,114^ Some phages are highly specific to certain bacterial strains, while others have broader ranges of host cells.^115^ After infection of host by phages, highly virulent phages often cause cell lysis (lytic cycle) while mild phages either lyse the host cell or keep the host cell alive and reproduce normally (lysogenic cycle).^116,117^

In summary, large-scale efforts are still required urgently in order to culture and catalog gut phages, which is much like the process of building collections and genome databases of bacterial strains in the human microbiome.^118^

### Is it pathogens?

Phages are regarded as potential human pathogens.^119^ This was probably first suggested with the genome of lambdoid phages that could encode Shiga toxins (Stx),^120^ which poses a severe and lethal threat to human health. Moreover, animal models revealed that phages may act as pathogens by affecting the gut bacterial communities and increasing the gut permeability.^121^ Besides, An increasing number of evidence indicates that human diseases, such as inflammatory bowel disease,^109,122^ diabetes,^123^ acquired immune deficiency syndrome,^124^ and colorectal cancer,^125^ are closely related to the gut viruses. Nevertheless, it is difficult to determine whether the changes in the gut virome are a cause or a result of those diseases. And the boundary between ‘normal’ and pathogenic virome is blurred because the same virus can be either a symbiont or a pathogen depending on the conditions such as the health status and development stage of the host. On the whole, the current knowledge of symbiotic viruses lags far behind that of pathogenic viruses.^126^

### Gut virome and alcohol-associated liver disease

The exact role of gut virome in the etiology of ALD is still unclear, as not only the complex pathogenesis of ALD but also our fragmented understanding of the gut viruses and limited relevant studies. In 2020, scientists systematically described an intestinal virome signature in AH patients for the first time. They observed an increased viral diversity in the stools of patients with ALD. In AH patients, *Escherichia-, Enterobacteria-*, and *Enterococcus* phages were over-represented and mammalian viruses such as *Parvoviridae* and *Herpesviridae* significantly increased. *Staphylococcus* phages and *Herpesviridae* were associated with severity and mortality of ALD.^127,128^ Above findings were consistent with previous research. In fecal samples, most detected *Herpesviridae* such as herpesvirus‐6^128,129^ and herpesvirus‐8^130,131^ are associated with the severity of ALD, and they could be classified into Epstein-Barr virus (EBV). EBV infection often causes liver inflammation, but the pathogenesis remains unknown.^129^ A retrospective study of patients with liver cirrhosis found that EBV-positive patients had higher Child-Pugh scores, more severe liver injury and a higher rate of chronic acute liver failure,^132^ which suggested the reactivation of EBV might contribute to the development of alcoholic hepatitis.

Gut virome may regulate the pathogenesis of ALD through multiple interactions with symbiotic bacteria.^133^ Phages, as the main component of the gut virome, are able to mediate bacterial cells lysis and regulate the abundance of bacteria. A recent study confirmed that the interaction between bacteria and phages in patients with cirrhosis and hepatic encephalopathy centered on *Streptococcus* species.^134^ Moreover, gut bacteria have access to additional genomes (such as antibiotic-resistance genes or bacterial virulence factors) transferred by phages, which will adjust bacterial virulence and adaptability and modify the composition of intestine microbiota.^135^

Furthermore, gut virome can interact with the host immune system directly. The intestinal immune system is mainly comprised of intestinal intraepithelial lymphocytes (IELs), lamina propria lymphocytes (LPLs), and Peyer’s patches (PPs),^136^ which could be important in the pathogenesis of ALD. Among them, commensal viruses can be recognized by retinoic acid-inducible gene 1 (RIG-1) signaling in antigen-presenting cells (APCs), and RIG-1 signaling can promote IL-15 production, prevent inflammation and tissue damage as well as maintain the homeostasis of IELs.^137^ Moreover, scientists found that the immune cells including CD4^+^ and CD8^+^ T cells increased in the germ-free mice treated with bacteriophages, and the increased abundance of bacteriophages can exacerbate intestinal colitis through toll-like receptor 9 (TLR9) and interferon-gamma (IFN-γ), indicating that phages can alter mucosal immunity to impact mammalian health.^138^ But, the mechanism of the phage recognition by the mucosal immune system needs more studies in animal models. Moreover, some studies on pathogenic enteroviruses, such as norovirus and rotavirus, have revealed several signaling pathways of intestinal recognition of viral nucleic acids.^139,140^ Thus, the gut virome either commensal or pathogenic viruses is a hot topic in host immune response.

To sum up, gut virome may promote the progression of ALD through interventions with the symbiotic bacteria and immune system^141^ . However, it is still unclear for patients with ALD what specific impacts of increased gut viruses are on the other microorganisms or the host body. We do not know yet whether liver inflammation or damage is caused by the virus itself, or its metabolites, or changes of other microbes induced by the altered gut virome. The studies of gut virome are just starting and more studies are needed to help us understand the correlations between the gut virus, the health of the host and liver diseases.

### Phage therapy of ALD

Recent animal studies creatively revealed the importance of phages in the treatment of ALD. According to a study in 2019,^142^ *Enterococcus faecalis* was significantly increased in fecal samples and the cytolysin-positive (cytolytic) *E. faecalis* correlated with the severity and mortality of AH patients. Then, researchers screened out bacteriophages that can lyse the cytolytic *E. faecalis* to treat mice transplanted with the gut microbiota of AH patients, which significantly reduced the levels of cytolysin in mice liver and attenuated alcohol-induced liver inflammation.

Editing intestinal microbes to treat or improve diseases related to microbial dysbiosis seems to be a quite promising direction. After all, antibiotics are not suitable for precise editing due to their broad spectrum, instead, some bacteriophages are highly specific to bacteria, suggesting a unique advantage of manipulating microbiota.^143,144^Certainly, the clinical application of phage therapy still needs the support of large-scale human data. In addition, the limitation of phage therapy may be due to its high specificity. Different patients need to deploy different phages in order to better efficacy, so its mass production-like antibiotics may be unrealistic in some extent. In the future, it is necessary to construct a huge phage bank, which will efficiently help us screen out concrete phages and make a personalized treatment within a short period of time.

## Gut archaeome

### Composition and distribution

Archaea were originally discovered and isolated from ecosystems with extreme conditions, including environments with high temperature, strong acid or base, and high ion concentration. However, with the continuous advancing of detection techniques, archaea had also been found in some mild environments such as the ocean ecosystems.^145–149^ Archaea are similar to bacteria in terms of shape, size, and genetic information expression including DNA replication, RNA transcription, and protein synthesis. Beside these similarities, there are also some obvious differences between archaea and bacteria. For instance, the archaeal cell walls do not contain peptidoglycans,^150^ and their cell membranes are composed of L-glycerol-ether/isoprenoid lipids, which are more stable and rigid than bacterial.^151^ Moreover, due to its special metabolic patterns, archaea can use sunlight, inorganic or organic substances as energy sources.^152^

The most abundant archaea in the human gastrointestinal tract are *Methanobacteriales* and *Methanomassiliicoccales*.^153^ The former, mainly consisting of *Methanobrevibacter smithii* and *Methanosphaera stadtmanae*,^154^ is identified as a keystone species that has a noticeable impact on the composition and function of other gut microbiome.^155,156^ The species of archaea are usually affected by various factors such as diet, environment, age, genetics, etc., and they are also related to non-archaea members of the host microbiota. Interestingly, the abundance of *M. smithii* was recently found to be stable over time, even after major changes in diet.^157^ In addition, the archaea present highly specific adaptability in the gastrointestinal tract, and they often evolve unique features such as modifications of cell surface (adhesin-like proteins (ALPs), glycans, bile salt hydrolases, and biofilm formation), which are not found in wide archaea, to escape the host-defense mechanism.^158^

Generally speaking, as an important part of the gastrointestinal microbiota of humans and animals, the role of archaea may be far more underestimated owing to the methodological shortcomings.^159^ For instance, most of the so-called “universal” 16S rRNA primers fail to picture the diversity of archaeal signatures and thus are unable to detect certain archaeal lineages in specific sample types.^160,161^ The archaea research is still in its infancy and the academics of microbiology lack relevant knowledge as well.

### Archaea and human diseases

Whether archaea are pathogens or not has always been a controversial topic.^158^ Some studies verified the change of archaeome in certain human diseases. For example, *M. smithii* was found in urinary tract infections.^161^ *M. oralis* was reported in periodontitis, and its quantity increased as the illness severity increased, but it was no longer present after healing, which highlighted the close association of *M. oralis* with the inflamed site.^162^ The abundance of *M. stadtmanae* increased three-fold in inflammatory bowel disease (IBD) patients when compared to healthy individuals, indicating this archaeon might be involved in pathologic process within the human gut.^163^

Metabolites produced by archaea such as methane can also affect the health status of the host. In dog models, methane can weaken gastrointestinal motility.^164^ In a population with irritable bowel syndrome (IBS), the subjects with the constipation-dominant disease (IBS-C) were shown to have a higher proportion of methane producers than individuals with the diarrhea-dominant disease (IBS-D).^165^

Furthermore, people have also found the interaction between archaea and the human immune system. When stimulating the immune system with *M. stadtmanae*,^154^ high levels of proinflammatory cytokines including interleukin and interferon were released.^166,167^ Only recently was it demonstrated that RNA from *M. stadtmanae* was a potent immune stimulator, and toll-like receptor 7 (TLR7) and TLR8 were identified as the involved pattern recognition receptors. Moreover, this molecular interaction induced TLR8-dependent NLRP3 inflammasome activation.^168^ The proinflammatory ability of archaea also varies from species to species. For example, *M. stadtmanae* is capable of inducing a stronger immune response than both *M. smithii* and *M. luminyensis*.^166,169^ There are some speculations attempting to explain the mild response induced by *M. smithii* stating that *M. smithii* has the capacity to produce glycans which is similar to those found in the gut,^170^ and thus may help *M. smithii* escape from the host immune system. In conclusion, understanding the molecular mechanisms of how archaea induce inflammation in the body is thus an important step in uncovering how such diseases might develop.

The impact of ALD on the composition of archaea is rarely reported. According to a recent research, the proportion of archaea among the healthy control group, the alcohol use disorder group, and the alcoholic hepatitis group was 0.6%. 0.3%, and 0.0% respectively, which indicated that the abundance of archaea was reduced as the disease progresses.^171^ However, it is not clear which archaea have modified, nor is it clear whether these changes are a result of or a cause for the disease. But considering the proinflammatory ability, it is worthwhile to study the interspecies communication between archaea and other gut microbes, the potential role of archaeal metabolites, and the relationship of archaea and chronic liver diseases.

## Conclusion

Although we have generally devoted significant efforts on studying the gut bacteriota, it is worth noting that non-bacterial communities also have potential functions. All these microbes will interact with the host and also with each other, in combination or alone, to influence the health and disease of the host. Eventually, we will unveil the role of non-bacterial microbiota in human with the remarkable development of technologies for the detection of fungi, viruses, and archaea. Furthermore, human microbiota investigations have now reached a critical inflection point. In the future, we need to move rapidly from mere description or correlation to causation, and ultimately to translation.^172^

To accomplish such a great mission, much work remains ahead of us. The first is the improvement of methodology. At present, there are three kinds of methods commonly used in studying intestinal microbiota: culture-dependent methods, culture-independent methods, and high-throughput sequencing (HTS). There are also several excellent literatures evaluating these methods in gut mycobiome,^173^ virome,^174^ and archaeome.^160^ Nevertheless, most research tools still have some defects. Culture methods are time-consuming and also susceptible to environmental factors. Scientists might misestimate the abundance of certain gut microorganisms owing to the incompleteness of annotated gene databases or improper primers. In addition, the amount of relic DNA in human gut remains unknown currently, and a few fungi DNA can even be detected after years of organism decomposition. Thus, we need to be more prudent in face of the results of DNA-based sequencing. The study of gut non-bacterial microbiota will be more efficient through establishing a uniform, accurate, and highly operable detection system for non-bacteriota. And ensuring that the microbiome from different regions, races, and species can be compared or referenced is highly expected in future work.

Secondly, it is necessary to expand the genomics, transcriptomics, and metabolomics data of fungi, viruses, and archaea in the existing database, so that future research can accurately and quickly identify the corresponding microbiota in multiple dimensions. Moreover, the study of microbiome cannot simply stop at the macroscopic appearance of the gut community composition changes, it should be conducted from bacterial species to specific strains, from cells to subcellular organs, from metabolites to molecular regulation. Study in such detail will provide a fresh and fundamental perspective for manipulating and editing the gut microbiota logically and efficiently in the future. Finally, the commensal microbiota in the intestine is an emerging and promising field that has aroused great interest from scientists, physicians and patients. With a deeper understanding and transformation of the gut-liver axis, the investments may be rewarded and the management of ALD patients will also be improved.

## Supplementary Material

Supplemental Material
