# Supplementary material for: Gut non-bacterial microbiota contributing to alcohol-associated liver disease
Source: Gut Microbes. 2021 Oct 28;13(1):1984122. doi: 10.1080/19490976.2021.1984122 (PMC8565833; doi:10.1080/19490976.2021.1984122)
Supplement: Supplemental Material [file KGMI_A_1984122_SM5717.docx]

**Supplementary Table 1** The research of gut mycobiome in alcohol-associated disease

| **Species** | **Comparison** | **Change of gut microbiota** | | **Impacts on ALD** | **Potential pathways** | **Method** | **Reference** |
| --- | --- | --- | --- | --- | --- | --- | --- |
|  |  | **increased** | **decreased** |  |  |  |  |
| **Fungi** | Chronic alcohol administration vs. normal control (in mice)  Alcohol-dependent patients (n=20) vs. healthy individuals (n=8) | *Humicola*; *Fusarium*; *Aspergillus*  *Candida* (*Candida albicans* is the highest abundance species) | *Candida*  *Epicoccum*; *Galactomyces*; *Debaryomyces*; *unclassified fungi* | Fungi and its products aggravate ethanol-associated liver disease with high levels of liver injury and hepatic steatosis in mice; Fungal products correlates with mortality of patients with alcoholic cirrhosis | 1,3-β-gulcan activates CLEC7A (also called dectin-1) signaling pathway and induce the secretion of IL-1β from Kupffer cells | qPCR and ITS sequencing | An-Ming Yang, et al. [73] |
|  | Alcoholic hepatitis (n=59) vs. non-alcoholic controls (n=11)  alcohol use disorder (n=15) vs. non-alcoholic controls (n=11) | *Candida*  *Candida*  *Aspergillus* | *Penicillium*; *Saccharomyces*; *Debaromyces*  *Penicillium*; *Saccharomyces*; *Debaromyces* | *Candida* is positively correlated with pericelluar fibrosis;  *Penicillium* is negatively correlated with inflammatory grade on liver biopsy | Not mentioned | ITS sequencing | Songja Lang, et al. [74] |
|  | Alcoholic hepatitis (n=81) vs. controls (n=9) | *Candida* species (especially *Candida* *glabrata*) | Not mentioned | Not mentioned | Not mentioned | Shotgun metagenomic sequencing | Bei Gao, et al. [171] |
| **Fungi** | Ethanol-fed mice vs. control diet-fed mice | *Meyerozyma*; *guilliermondii* | Not mentioned | *M. guilliermondii* aggravates fat accumulation and inflammatory injury of liver | Gut fungi-induced hepatic PGE2 exacerbates alcoholic hepatic steatosis | Culture and ITS sequencing | Shanshan Sun, et al.[85] |
|  | Ethanol-fed mice vs. healthy mice | *Saccharomycetaceae*; *Candida_f_Debaryo*;*mycetaceae*; *fungal_sp* | Not mentioned | Not mentioned | Dectin-1/IL-1β signaling pathway and the canonical NLRP3/ASC/pro-caspase-1 signaling pathway | ITS sequencing | Jiadi Wu, et al. [77] |
|  | Alcoholic hepatitis (n=91) vs. controls (n=11)  Alcoholic hepatitis (n=91) vs. Alcohol use disorder (n=42)  Wide type *C. albicans* treated mice vs. PBS treated mice (both ethanol feeding)  ECE1-deletion *C. albicans* treated mice vs. Wide type *C. albicans* treated mice | *Candida albicans* (ECE1-positive *C. albicans* are more abundant in the AH group)  Not mentioned | Not mentioned  Not mentioned | ECE1-positive *Candida albicans* encoding Candidalysin exacerbates ethanol-induced liver injury, steatosis and inflammation | Independent of dectin-1 signaling pathway | Culture;  qPCR;  ITS sequencing | Huikuan Chu, et al. [87] |
|  | Alcohol use disorder (n=66) vs. controls (n=18) | *Candida* (especially *Candida albicans* and *Candida zeylanoides*); *Debaryomyces*; *Pichia*; *Kluyveromyces*; *Issatchenkia* | *Aspergillus* | Not mentioned | Not mentioned | ITS sequencing | Phillipp Hartmann, et al.[76] |

AH, alcohol-associated hepatitis; ALD, alcohol-associated liver disease; ASC, apoptosis-associated speck-like protein containing a CARD; ECE1, the extent of cell elongation 1; CLEC7A, C-type lectin domain family 7 member A; ITS, internal transcribed spacer regions; qPCR, quantitative polymerase chain reaction; PGE2, prostaglandin E2; NLRP3, NOD-like receptor family pyrin domain containing 3

**Supplementary Table 2** The research of gut virome and archaeome in alcohol-associated disease

| **Species** | **Comparison** | **Change of gut microbiota** | | **Impacts on ALD** | **Potential pathways** | **Method** | **Reference** |
| --- | --- | --- | --- | --- | --- | --- | --- |
|  |  | **increased** | **decreased** |  |  |  |  |
| **viruses** | Alcoholic hepatitis (n=89) vs. healthy individuals (n=17) | *Proteobacteria* and *Firmicutes* phages including *Lactobacillus*-, *Escherichia*-, *Enterobacteria*, and *Enterococcus* phages; mammalian viruses like *Parvoviridae* and *Herpesviridae* | *Sk1virus*; *Siphoviridae* | *Herpesviridae* might contribute to the development of AH | Not mentioned | metagenomic sequencing and real-time qPCR | Lu Jiang, et al. [127] |
|  | Mice treated with phages that target cytolytic *E. faecalis* vs. controls | Not mentioned | Not mentioned | The phages against cytolytic *Enterococcus faecalis* alleviated ethanol-induced liver injury and steatosis | Not mentioned | Bacteriophages isolation and amplification; whole-genome sequencing | Yi Duan, et al. [142] |
| **archaea** | Controls (n=9), patients with alcohol use disorder (n=41) and alcoholic hepatitis (n=81) | The percentage of archaea in the three groups are 0.6%, 0.3% and 0.0% respectively; The exact genera are not mentioned | | Not mentioned | Not mentioned | 16S rRNA sequencing | Bei Gao, et al. [171] |
